# Integrating Planetary Health Into Interprofessional Education: A Scoping Review

**DOI:** 10.7759/cureus.103662

**Published:** 2026-02-15

**Authors:** Abdulqadir J Nashwan, George V Joy, Nabila Chaabna

**Affiliations:** 1 Nursing and Midwifery Research Department, Hamad Medical Corporation, Doha, QAT

**Keywords:** collaborative learning, global health, interdisciplinary education, interprofessional education, one health, planetary health

## Abstract

Planetary health underscores the relationship between human well-being and the environment. It focuses on how climate change, pollution, and biodiversity loss affect people’s health. Integrating this concept into interprofessional education (IPE) is vital for preparing health professionals to address these issues. This scoping review aimed to map the existing literature on IPE initiatives in planetary health, examine outcomes, and identify research gaps. It also aimed to inform educators, policymakers, and researchers about effective strategies and areas that require further investigation. Following established scoping review methodology, comprehensive searches were conducted in PubMed, Scopus, Embase, and Cumulative Index to Nursing and Allied Health Literature (CINAHL) databases using specified search terms. Studies from 2019 to 2024 were included, reflecting recent developments in IPE on planetary health. Two reviewers independently performed screening and data extraction, with conflicts resolved through discussion. A total of 32 studies met the inclusion criteria, showcasing diverse educational approaches across medical education, public health, and nursing. The review identified varying levels of curriculum integration, impacts on knowledge and confidence, and challenges such as a lack of standardization, resource limitations, and barriers to interdisciplinary collaboration. Findings were categorized by discipline, methodology, country of origin, and educational focus, providing insights into the evolving landscape of health professional education in response to planetary health challenges. The synthesis demonstrates the importance of integrating planetary health into health professional curricula to effectively address global environmental changes. Key findings illustrate the diversity of educational approaches and underscore the need for standardized frameworks, effective resource allocation, and enhanced interdisciplinary collaboration to strengthen educational initiatives.

## Introduction and background

Planetary health underscores the interdependencies between human health and the state of our planet's natural systems [1]. It addresses the multifaceted impacts of environmental changes such as climate change, pollution, and biodiversity loss on human health [2].

Although both One Health and planetary health address the interconnectedness of health and the environment, their scopes and approaches differ. One Health traditionally focuses on the direct interfaces between human, animal, and environmental health, emphasizing zoonotic diseases, antimicrobial resistance, and ecosystem health. It typically involves collaboration among veterinary, medical, and environmental sciences to optimize health outcomes by recognizing the connections among people, animals, and their shared environment [3]. In contrast, planetary health adopts a broader perspective, encompassing these direct interfaces and the systemic impacts of human activity on the planet's natural systems, including climate change and biodiversity. Planetary health advocates for sustainable development that ensures the health of human civilization and the natural systems on which it depends [4].

Interprofessional education (IPE) involves collaborative learning across various professional disciplines, particularly in healthcare, to enhance teamwork and improve patient outcomes [5]. Integrating planetary health into IPE is crucial for preparing health professionals to address the complex, interconnected challenges posed by environmental change [6]. Recent literature highlights the importance of incorporating sustainability and environmental health into health professional education [7-9]. However, a comprehensive synthesis of IPE initiatives explicitly focused on planetary health remains limited. This gap in the literature underscores the need for a scoping review to map existing educational interventions, identify their outcomes, and highlight areas that require further research.

The primary objective of this review was to assess the extent of the literature on IPE initiatives focused on planetary health, examining their outcomes and impacts. By mapping the current landscape of IPE in this context, the review provides a comprehensive overview to inform educators, policymakers, and researchers about effective strategies while highlighting gaps for future investigation.

IPE is crucial for promoting collaboration among healthcare professionals and addressing complex planetary health issues. Understanding the existing educational strategies and their effectiveness can guide the development of more robust and impactful IPE programs. This scoping review, therefore, aimed to fill a critical gap in the literature by synthesizing evidence on IPE initiatives related to planetary health. The findings offer valuable insights into how health professional education can evolve to meet the challenges posed by global environmental changes, ultimately contributing to the well-being of the entire planet.

## Review

Methods

This scoping review follows the framework proposed by Arksey and O’Malley [10], enhanced by Levac et al. [11], and adheres to the Joanna Briggs Institute (JBI) methodology for scoping reviews [12]. The review encompasses both published and unpublished studies and was conducted using a comprehensive and systematic search strategy.

Study Identification

The review methods were designed to identify and examine the existing literature on IPE and planetary health. A scoping review methodology was selected because the literature was expected to be heterogeneous in study designs and publication types. The Preferred Reporting Items for Systematic Reviews and Meta-Analyses Extension for Scoping Reviews (PRISMA-ScR) was followed as the reporting guideline for this study [13].

Electronic databases searched included PubMed, Scopus, Embase, and Cumulative Index to Nursing and Allied Health Literature (CINAHL). Search terms were carefully selected to retrieve studies addressing both IPE and planetary health, using the following terms: “interprofessional education” OR “interprofessional” AND “education” OR “interprofessional education” AND “planetary health” OR “planetary” AND “health”. The initial search was conducted between June 30 and July 7, 2024. Scholarly and research articles meeting the predefined criteria were retrieved from each database.

Study Selection Criteria

Articles published between 2019 and 2024 were included to capture the evolution and recent developments of IPE related to planetary health. An interprofessional research team with backgrounds in public health, education, nursing, and health sciences conducted the search and screening processes.

Inclusion criteria comprised studies that presented planetary health within the context of IPE, conducted in any educational setting (including clinical, classroom-based, interprofessional, and continuing education) and in any country.

Exclusion criteria included publications not written in English, studies focused on school-level education, articles addressing health consequences without an educational component, and publications without a clear description of IPE.

Data Extraction and Synthesis

Titles and abstracts of retrieved records were screened for relevance according to the inclusion criteria. A total of 63 articles were identified through database and hand searches. After removing 17 duplicates, 46 titles and abstracts were screened. Two articles were excluded at this stage, and 44 full-text articles were assessed for eligibility.

**Figure 1:**
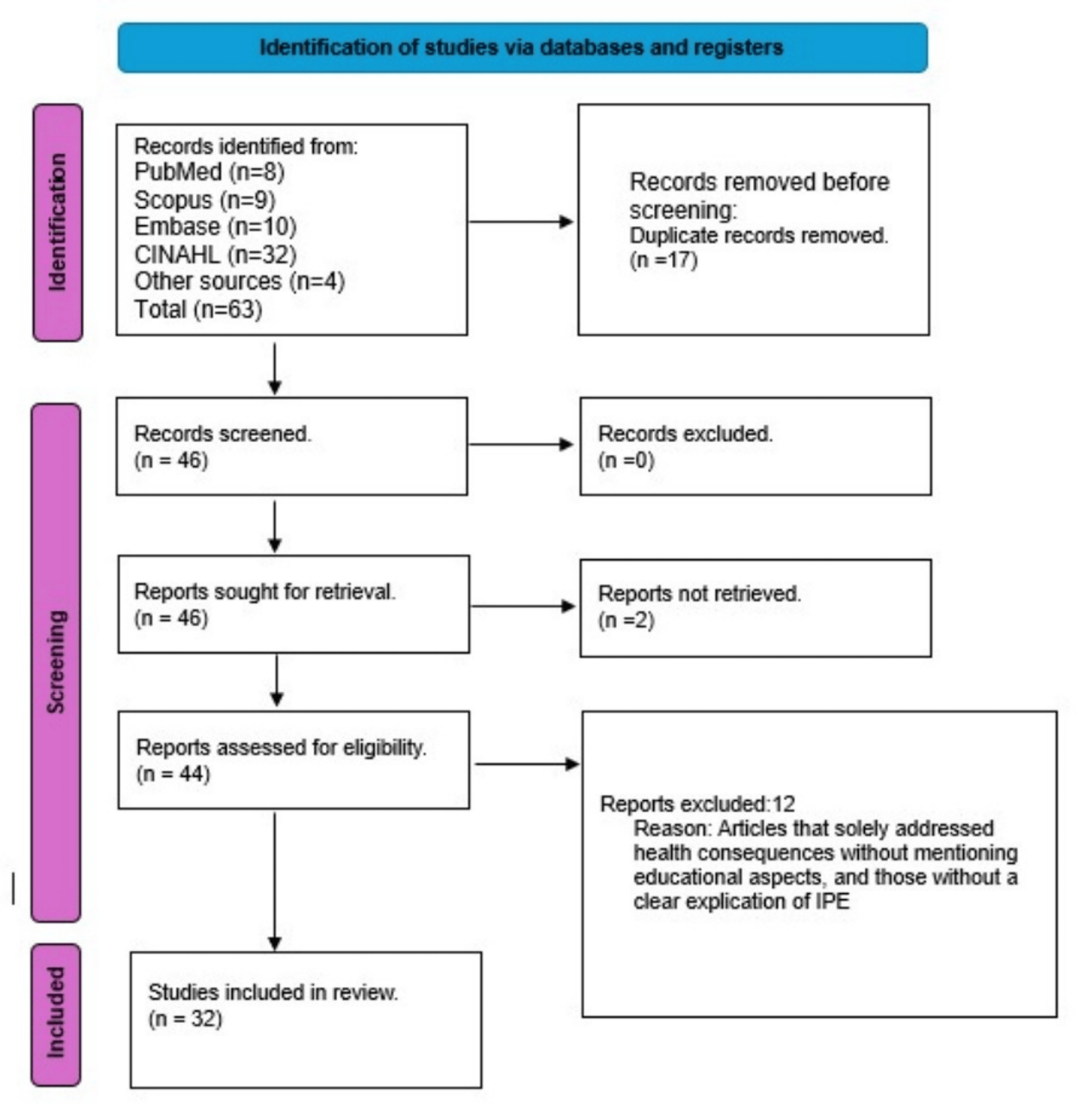
PRISMA-ScR flowchart PRISMA-ScR: Preferred Reporting Items for Systematic Reviews and Meta-Analyses Extension for Scoping Reviews; CINAHL: Cumulative Index to Nursing and Allied Health Literature; IPE: Interprofessional education

Figure 1 presents the modified Preferred Reporting Items for Systematic Reviews and Meta-Analyses (PRISMA) flow diagram consistent with the PRISMA-ScR checklist, outlining the processes of identification, screening, eligibility assessment, and inclusion [13].

Following full-text review, 12 articles were excluded, leaving 32 studies for the final analysis [8,14-44]. Two reviewers (GVJ and NC) independently and without bias reviewed all articles, with any disagreements resolved through discussion with AJN. Additional relevant studies were identified by examining the reference lists of included articles.

Data were extracted using a customized Excel extraction form (Microsoft Corp., Redmond, USA). Two reviewers (GVJ and NC) independently extracted data from each study, and discrepancies were resolved by consensus. The findings were discussed with the wider manuscript team to confirm agreement on inclusion and interpretation.

Thematic analysis was employed to synthesize the data. Two authors (GVJ and NC) independently coded and interpreted the findings and then met to agree on major themes. Themes were refined iteratively and categorized by discipline, study methodology, country of origin, and interprofessional focus. Ethical approval was not required as no primary data collection or human subjects were involved.

Results

The findings from this scoping review highlight essential insights and contributions of IPE regarding the pivotal roles various health disciplines play in addressing planetary health. Each discipline contributes distinct perspectives and expertise, underscoring the importance of collaboration in addressing the multifaceted challenges of planetary health.

**Figure 2:**
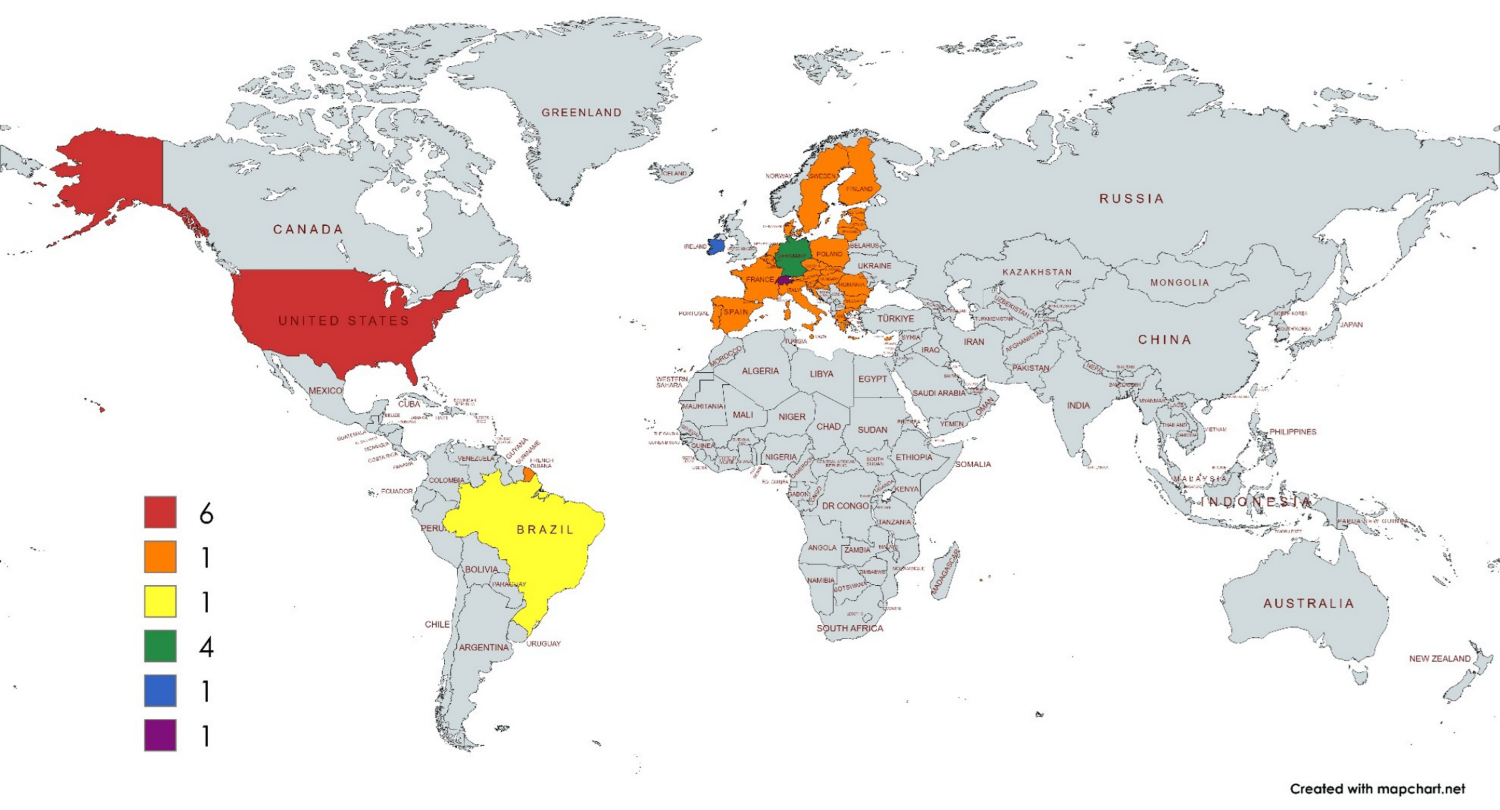
Countries of origin of included studies Unspecified/international papers = 18, USA = 6, Germany = 4, Ireland = 1, Brazil = 1, Switzerland = 1, Europe = 1 Map created using MapChart (www.mapchart.net).

**Figure 3:**
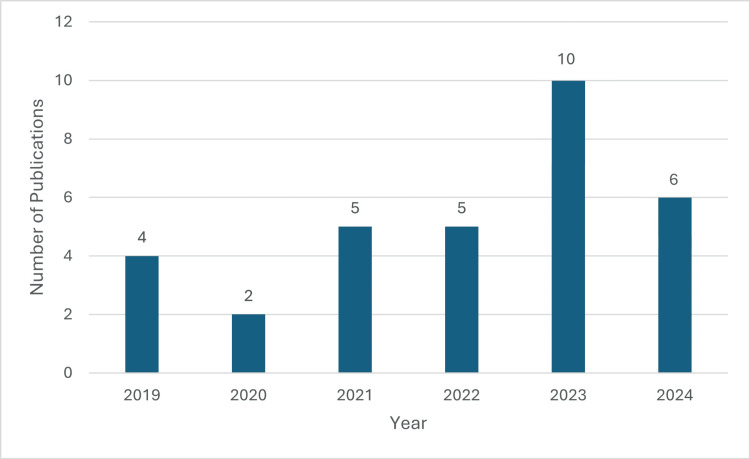
Number of publications by year Studies are categorized based on the year first published online.

The types of papers included in this scoping review (n = 32) are summarized in Table 1, while the countries of origin of the included studies are presented in Figure 2. The distribution of publications by year is illustrated in Figure 3.

**Table 1:** Types of papers included 'Others' refers to frameworks or technical reports.

Label	Number (n)
Consensus statement	1
Original research	8
Correspondence/short communication/commentary/discussion paper	10
Review	5
Framework/theory/course design	4
Book chapter	1
Others	3

Table 2 provides a detailed summary of the included studies, including author, year of publication, discipline, study purpose, and key findings relevant to the review question.

**Table 2:** Results summary IPE: Interprofessional education; CME: Continuing medical education; SDGs: Sustainable Development Goals

Reference	Author and Year	Country	Aim	Key Findings	Type
[14]	Shaw et al., 2021	Europe	To integrate planetary health principles into medical education.	Outlined essential reforms aligned with the SDGs to prepare future healthcare professionals.	Consensus statement
[15]	Schwienhorst-Stich et al., 2023	Germany	An overview of initiatives promoting planetary health education.	Demonstrated growing national initiatives spanning curricula and interdisciplinary projects.	Short communication
[16]	Flägel et al., 2023	Germany	To integrate planetary health into digital teaching qualifications.	Digital teaching platforms enhanced interdisciplinary education on planetary health.	Project report
[17]	Garman et al., 2023	USA	To develop a practice-based planetary health education approach.	Immersive interdisciplinary learning improved understanding and leadership skills.	Course design
[18]	Nordrum et al., 2022	Ireland	To examine planetary health education in medical curricula.	Identified the need for more standardized curricular integration.	Original research
[19]	Trudel et al., 2024	USA	To evaluate an outdoor course on planetary health.	Promoted holistic health understanding and learner well-being.	Original research
[20]	Shafto et al., 2023	USA	To assess experiential education in food systems and nutrition.	Significantly improved learners’ knowledge and confidence.	Original research
[21]	Gepp et al., 2023	Germany	To evaluate the Planetary Health Academy virtual series.	Enhanced engagement and interdisciplinary dialogue through virtual learning.	Original research
[22]	Liu et al., 2022	USA	To explore student perspectives on a co-created climate curriculum.	Increased engagement, relevance, and preparedness.	Original research
[23]	Jacobsen et al., 2024	USA	To compare four United States planetary health curricular models.	Diverse models shared a common sustainability-oriented goal.	Theory paper
[24]	Visser et al., 2024	Global	To review characteristics of planetary health in medical education.	Identified global trends, gaps, and curriculum innovations.	Scoping review
[25]	Hess and Rihtman, 2023	Global	To integrate planetary health into occupational therapy education.	Emphasized skill-based approaches, bridging theory and practice.	Theory paper
[26]	LeClair, 2021	Global	To examine kincentric awareness in planetary health education.	Advocated holistic, non-anthropocentric educational approaches.	Discussion paper
[27]	Guzmán et al., 2021	Global	To develop a planetary health education framework.	Identified five foundational domains for planetary health learning.	Framework
[28]	Levett-Jones et al., 2025	Global	To achieve consensus on nursing competencies in planetary health.	Emphasized sustainability, advocacy, and environmental stewardship.	Delphi study
[29]	Demorest et al., 2024	USA	To examine the integration of climate change in nursing education.	Highlighted effective strategies improving climate-health literacy.	Original research
[8]	McKinnon et al., 2022	Global	To examine IPE's role in addressing climate change impacts.	IPE enhanced collaboration and preparedness across professions.	Scoping review
[30]	Rieser et al., 2023	Switzerland	To integrate planetary health into postgraduate and CME training.	Improved awareness and preparedness among healthcare professionals.	Discussion paper
[31]	Rosa et al., 2021	Global	To examine nurses’ and midwives’ roles in achieving the SDGs.	Highlighted leadership roles in sustainable development.	Discussion paper
[32]	Potter, 2021	Global	To present a planetary health framework for nursing education.	Positioned planetary health as essential to nursing practice.	Framework
[33]	Potter, 2019	Global	To advocate for planetary health integration in nursing education.	Emphasized nurses’ role in environmental health advocacy.	Short communication
[34]	MacKenzie-Shalders et al., 2024	Global	To identify guides and frameworks for planetary health education.	Highlighted adaptable tools and diverse educational strategies.	Review
[35]	Baena-Morales and Fröberg, 2023	Global	To propose practical recommendations for education integration.	Offered actionable guidance for curriculum development.	Discussion paper
[36]	McKimm and McLean, 2020	Global	To promote eco-ethical leadership in health professions education.	Emphasized leadership development for sustainability.	Commentary
[37]	Barna et al., 2020	Global	To explore health education in the Anthropocene era.	Highlighted ecological determinants of health.	Discussion paper
[38]	Godinho et al., 2019	Global	To examine academic policy debates on climate leadership.	Demonstrated policy debate as a catalyst for climate action.	Correspondence
[39]	Gilbertson et al., 2019	Global	To promote interdisciplinary planetary health collaboration.	Highlighted partnerships addressing global health challenges.	Commentary
[40]	Walpole et al., 2019	Global	To integrate planetary health into clinical education.	Outlined strategies for sustainable clinical training.	Discussion paper
[41]	Zandavalli et al., 2024	Brazil	To evaluate a systems approach in planetary health education.	Systems thinking improved the understanding of health–environment interconnections.	Original research
[42]	Albrecht et al., 2023	Germany	To assess climate-specific health literacy among health professionals.	Identified need for targeted education to improve climate-health literacy.	Original research
[43]	Levin-Zamir and Nogueira, 2022	Global	To introduce innovative pedagogies in health promotion education.	Experiential, digital, and interprofessional pedagogies enhanced learning.	Book chapter
[44]	Moloo et al., 2022	Global	To map the integration of planetary health in medical education.	Identified gaps and opportunities in curricula and training.	Scoping review

This scoping review examining IPE and planetary health included studies from a range of educational and professional contexts. Most studies focused on medical education (n = 22), followed by nursing education (n = 5). Additional contributions came from public health (n = 2), general education (n = 2), and combined medical and nursing education (n = 1). This distribution reflects a strong emphasis on medical education, with comparatively fewer studies exploring interprofessional or cross-disciplinary educational initiatives involving nursing and other health professions.

Discussion

This scoping review aimed to fill a critical gap in the literature by synthesizing evidence on IPE initiatives that address planetary health. The findings from the included studies demonstrate an evolving response within health professional education to the challenges posed by global environmental changes. Collectively, the evidence highlights growing recognition of the need to embed planetary health concepts across health disciplines and to foster collaborative, interdisciplinary learning environments.

The results indicate that although planetary health is increasingly acknowledged as a core educational priority, its integration into IPE remains uneven. Many initiatives remain discipline-specific, particularly in medical education, while fewer studies explicitly adopt interprofessional frameworks. This imbalance suggests missed opportunities to leverage IPE to address complex, system-level environmental health challenges that require coordinated, cross-disciplinary responses.

Diverse Approaches to Planetary Health Education

The reviewed studies demonstrate a wide range of approaches to integrating planetary health into health professional education. The Association for Medical Education in Europe (AMEE) consensus statement outlines comprehensive reforms needed within medical education, emphasizing global collaboration and sustainability-oriented curricula [14]. National initiatives in Germany further reinforce these objectives by systematically incorporating planetary health principles into medical training programs [15].

Curricular innovations such as elective courses and interdisciplinary projects have also emerged as effective strategies. For example, Flägel et al. introduced planetary health as a central curricular theme through structured teaching sessions and multidisciplinary collaboration [16]. Similar approaches across different countries reveal varied levels of curricular integration, reflecting diverse educational contexts, institutional priorities, and resource availability [16,17].

Impact on Knowledge and Confidence

Earlier nursing scholarship also emphasized the need to integrate planetary health into professional education, highlighting positive effects on students’ awareness and preparedness [19]. Additionally, virtual learning initiatives, such as the Planetary Health Academy, demonstrated significant improvements in participants’ knowledge and engagement, underscoring the potential of accessible, scalable educational formats for planetary health education [21].

Several studies evaluated the educational impact of planetary health interventions on learners’ knowledge, confidence, and engagement. Shafto et al. demonstrated that experiential learning approaches, particularly hands-on culinary and food systems education, significantly improved learners’ understanding of planetary health concepts and their confidence in applying this knowledge in practice [20].

Curriculum Integration and Skills Development

Effective planetary health education requires not only conceptual knowledge but also practical skills applicable to clinical and public health practice. Jacobsen et al. identified commonly used curricular frameworks in United States institutions, underscoring the absence of a standardized national approach [23]. This variability may limit the transferability and consistency of educational outcomes across settings.

Other scholars emphasized translating planetary health theory into practice by developing competencies that bridge the gap between knowledge and action. Hess and Rihtman highlighted the importance of skill-based training for applying planetary health principles in occupational therapy practice [25]. At the same time, LeClair emphasized kincentric awareness as a means of strengthening learners’ practical engagement with environmental health concepts [26]. A recent scoping review further identified critical gaps in skills-based planetary health education and recommended greater emphasis on interdisciplinary and transdisciplinary learning approaches [24].

Challenges in Integrating IPE Into Planetary Health

Despite increasing momentum, several challenges persist in embedding planetary health within IPE frameworks.

Lack of standardization: There is a notable lack of standardized curricular frameworks for planetary health education across institutions and countries. The heterogeneity identified by Jacobsen et al. highlights the absence of unified educational standards, which may undermine consistency and effectiveness [23].

Resource limitations: Implementing comprehensive planetary health curricula requires substantial institutional resources, including trained faculty, teaching materials, and administrative support. Many programs face constraints in these areas, which can limit the scope and quality of educational delivery. Resource-related barriers have been consistently identified as a significant obstacle to sustained curricular integration [23].

Engagement and interdisciplinary collaboration: Although interdisciplinary collaboration is central to planetary health education, achieving meaningful engagement across disciplines remains challenging. Siloed educational structures, differences in curricular priorities, and uneven faculty expertise hinder the implementation of robust interprofessional initiatives. National efforts in Germany illustrate both progress and persistent structural barriers to interdisciplinary engagement [21].

Strengths

This scoping review contributes to the literature on planetary health and IPE. We systematically searched multiple databases, ensuring comprehensive coverage of the relevant literature from various disciplines. The review encompasses articles published between 2019 and 2024, capturing the most recent ideas, trends, and developments in the field and ensuring the relevance of its findings to current educational practices. Additionally, adherence to established scoping review frameworks, such as the JBI methodology and PRISMA-ScR guidelines, increases the rigor and transparency of the review process and enhances its credibility.

Limitations

This scoping review has several limitations that should be acknowledged. First, the literature search was conducted between June 30 and July 7, 2024; therefore, studies published after this period were not captured. Given the rapidly evolving nature of planetary health education, relevant contributions from the latter half of 2024 and beyond may have been excluded. Future updates of this review would strengthen the currency and comprehensiveness of the findings.

Second, the included studies are geographically concentrated, primarily originating from Europe and the United States, with limited representation from low- and middle-income countries. This geographic imbalance may limit the global generalizability of the conclusions. Additionally, only English-language publications were included, which may have excluded relevant research published in other languages and contributed to language bias.

Third, as a scoping review, this study included diverse publication types (e.g., original research, reviews, discussion papers, and frameworks). While this heterogeneity is consistent with scoping review methodology, it may limit the depth of synthesis and comparability across studies. Finally, we acknowledge the need for careful alignment between cited references and the specific claims made in the manuscript.

## Conclusions

The integration of planetary health into IPE is no longer optional but essential for preparing future health professionals to address complex, climate-driven health challenges. This scoping review maps the global landscape of educational efforts, demonstrates growing momentum across disciplines, and highlights persistent gaps in standardization, competency development, and interdisciplinary coordination.

To advance the field, researchers should prioritize developing and validating IPE-specific planetary health competency frameworks and evaluating measurable educational and practice outcomes. Educators should move beyond conceptual awareness toward skill-based, practice-oriented modules that embed systems thinking, sustainability principles, and cross-disciplinary collaboration into core curricula. Policymakers and institutional leaders should support this transition by establishing clear curricular standards, mandating integration where appropriate, and allocating sustainable funding to support faculty development and interprofessional initiatives. Collectively, a coordinated effort across research, education, and policy is required to transform planetary health from an emerging educational theme into a structured, competency-driven pillar of health professional training.
