# Correction: Integrating Planetary Health Into Interprofessional Education: A Scoping Review

**DOI:** 10.7759/cureus.c402

**Published:** 2026-02-17

**Authors:** Abdulqadir J Nashwan, George V Joy, Nabila Chaabna

**Affiliations:** 1 Nursing and Midwifery Research Department, Hamad Medical Corporation, Doha, QAT

This article has been corrected to replace Figure 3 as the original version included incorrect totals.

